# Incorporating Molecular Classification When Stratifying the Survival Risk of Patients with High-Grade Endometrial Carcinomas

**DOI:** 10.3390/jcm12020530

**Published:** 2023-01-09

**Authors:** Liju Zong, Shengwei Mo, Zezheng Sun, Zhaohui Lu, Jie Chen, Shuangni Yu, Yang Xiang

**Affiliations:** 1 Department of Gynecologic Oncology, Peking Union Medical College Hospital, Chinese Academy of Medical Sciences, Beijing 100730, China; 2 Department of Pathology, Peking Union Medical College Hospital, Chinese Academy of Medical Sciences, Beijing 100730, China; 3 National Clinical Research Center for Obstetric & Gynecologic Diseases, Beijing 100730, China

**Keywords:** endometrial cancer, molecular subtypes, prognosis, high-grade endometrial carcinoma

## Abstract

**Simple Summary:**

Assessing survival risk in patients with high-grade endometrial carcinomas has remained challenging. The aim of our retrospective study was to investigate the distribution of molecular subtypes and assess their prognostic role in patients with high-grade endometrial carcinoma. We found that patients with different molecular subtypes (but not different histotypes) had distinct survival times. When incorporating the four molecular classifications into the stratification model, 52 patients (15.5%) who had originally been stratified without taking these classifications into account switched risk groups, with 40 (11.9%) shifting to a lower risk for having a *POLE* mutation and 12 (3.6%) shifting to a higher risk owing to p53-mutant status. Our findings suggest that incorporating molecular risk can improve patient risk stratification and enhance treatment planning.

**Abstract:**

Assessing survival risk in patients with high-grade endometrial carcinomas has remained challenging. We aimed to investigate the distribution of molecular subtypes and assess their prognostic role in a large cohort of 355 patients with high-grade endometrial carcinoma. Molecular classification was determined using DNA polymerase epsilon (*POLE*) sequencing as well as immunohistochemical staining for p53 and mismatch repair (MMR) proteins. Endometrial carcinomas were stratified into four subtypes: *POLE* ultramutated, MMR-deficient, non-specific molecular profile (NSMP), and p53-mutant. This study included 177 and 178 patients with endometrioid and non-endometrioid carcinomas, respectively. Forty-two patients (11.8%) were categorized as *POLE* ultramutated, 106 (29.9%) as MMR-deficient, 128 (36.1%) as p53-mutant, and 79 (22.2%) as NSMP. Patients of different molecular subtypes had distinct survival times; molecular classification, but not histotype, was significantly associated with survival outcomes. When incorporating molecular classification into the stratification model, 52 patients (15.5%) switched risk groups, with 40 (11.9%) shifting to a lower risk for having a *POLE* mutation and 12 (3.6%) shifting to a higher risk owing to p53-mutant status. Molecular classification may provide more accurate prognostic information among patients with high-grade endometrial carcinomas and improve their stratification for purposes of clinical management.

## 1. Introduction

High-grade endometrial carcinoma refers to a heterogeneous group of malignancies that include International Federation of Gynecology and Obstetrics (FIGO) grade 3 endometrioid endometrial carcinoma (EEC), serous carcinoma, clear cell carcinoma, undifferentiated/dedifferentiated endometrial carcinoma (UEC/DEC), and carcinosarcoma [1,2]. Patient management and therapeutic strategies are governed by such morphological classifications. However, previous studies have shown that interobserver diagnostic agreement when histotyping high-grade endometrial carcinoma, including among specialist gynecologic pathologists, is suboptimal [3,4]. Additionally, some data suggest that the prognostic value of histological subtypes is questionable among patients with high-grade endometrial carcinomas. Some studies have shown that the survival of patients with serous carcinoma is poorer than that of patients with grade 3 EEC [5,6], while others indicate that the survival outcomes of these two groups of patients are comparable [7,8,9]. These limitations complicate the identification of patients who are at a high risk of recurrence as well as the development of individualized treatments. Therefore, there remains a critical need for improving and refining these risk groups among patients with high-grade endometrial carcinoma, and to further stratify their risks of recurrence.

In 2013, The Cancer Genome Atlas research network performed an integrated genomic and transcriptomic analysis of 373 endometrial carcinomas, including EEC and serous carcinoma, and classified them into four molecular subtypes with distinct prognoses: DNA polymerase epsilon (*POLE*) ultramutated (*POLE*mut) with an excellent prognosis, microsatellite instability (MSI) hypermutated with an intermediate prognosis, copy number-high with the worst prognosis, and copy number-low with an intermediate prognosis [10]. Based on the assessment of surrogate markers that are commonly measured in clinical practice, endometrial carcinomas are separately classified into four molecular subgroups that are analogous to the aforementioned classifications: *POLE*mut, mismatch repair (MMR)-deficient (MMRd), p53-mutant (p53mut), and tumors exhibiting none of these alterations that are referred to as having a non-specific molecular profile (NSMP), respectively [11]. Recently, such molecular classifications have been investigated in patients with grade 3 EEC [12,13] and other high-grade endometrial carcinomas such as clear cell carcinomas [14], carcinosarcomas [15], and UECs/DECs [16]. However, these studies focused on a single histological type, and no comparisons of these molecular classifications between patients with different types of high-grade endometrial carcinoma have been performed to date.

This study aimed to investigate these molecular subtypes and assess the prognostic utility of a clinically applicable molecular classification system in a large cohort of patients with high-grade endometrial carcinomas.

## 2. Materials and Methods

### 2.1. Patient Selection and Histopathological Review

We retrieved the records of patients diagnosed with grade 3 or 2–3 EEC and non-EEC (serous carcinoma, clear cell carcinoma, UEC/DEC, carcinosarcoma, and mixed carcinoma) between June 2010 and December 2018 at the Peking Union Medical College Hospital (Beijing, China). We obtained the original diagnoses from the archived hysterectomy specimen data and matched the curettage samples when available. All hematoxylin and eosin-stained slides were reviewed by two gynecological pathologists (S.Y. and Z.L.), and the histological type and grade of each tumor were confirmed based on morphological features. Each pathologist was blinded to both the original diagnosis and the other’s interpretation. Discrepant cases were examined under a multi-head microscope by three pathologists (S.Y., Z.L., and J.C.) to arrive at a consensus. This study was approved by the Institutional Review Board (JS-3230); informed consent was not required owing to its retrospective nature.

### 2.2. POLE Mutation Analysis

As described previously [17,18], DNA extracted using the QiaAMP DNA micro kit (QIAGEN Ltd., Manchester, UK) was used as a polymerase chain reaction template to amplify *POLE* exons 9–14; 150–200 bp products were then amplified using 100 ng of DNA extracted from formalin-fixed, paraffin-embedded samples. Sequencing was performed using BigDye version 3.1 terminator cycle sequencing chemistry on an ABI 3730 DNA analyzer (Applied Biosystems Inc., Foster City, CA, USA). All validated *POLE* mutations (P286R, V411L, S297F, A456P, and S459F) were subjected to bidirectional Sanger sequencing twice.

### 2.3. Tissue Microarray Construction, Immunohistochemistry, and Assessment

Representative areas of the tumor tissues were marked on hematoxylin and eosin-stained slides and sampled for tissue microarray (TMA) blocks. TMAs with duplicate 2 mm cores per case were constructed using a tissue arrayer (MiniCore, Mitogen, Hertford, UK). Immunohistochemistry was performed using our previously described laboratory protocol [19,20,21,22,23,24]. Briefly, 4 μm TMA serial sections were deparaffinized and subjected to heat-induced epitope retrieval using 10 mM sodium citrate (pH 6.0) at 95 °C for 20 min. Endogenous peroxidase activity was quenched using a 0.3% hydrogen peroxide solution. TMA sections were incubated with primary antibodies against p53 and the MMR-related proteins MSH2, MSH6, MLH1, and PMS2. Stromal and inflammatory cells served as internal controls for MMR and p53, while the same tissues probed with isotype-matched antibodies were used as negative controls.

### 2.4. Molecular Subgrouping

A tumor that exhibited complete loss of nuclear expression of any of the MMR proteins (MLH1, PMS2, MSH2, and/or MSH6) was considered MMRd, whereas that in which all four MMR proteins were detected (in the presence of an intact internal control) was considered MMR-proficient. Moreover, p53 mutation type was determined based on the presence of intense and diffuse nuclear staining, a complete absence of nuclear staining, or significant cytoplasmic staining in the presence of variable nuclear staining; wild-type expression was defined as weak and heterogeneous nuclear staining. As described previously [23,25], endometrial carcinomas were categorized into the following molecular subgroups: *POLE*mut (tumors with pathogenic variants in the exonuclease domain of *POLE*), MMRd (tumors with MMRd in the absence of *POLE* mutations), p53mut (tumors with mutation-type p53 staining in the absence of *POLE* mutations or MMRd), and NSMP (lacking any *POLE*, MMR, or p53 alterations).

### 2.5. Statistical Analysis

The χ^2^ test was used to determine associations between categorical variables. Relapse-free survival (RFS) was defined as the interval between the date of surgery and that of the detection of the first local, regional, and/or distant relapse. Disease-specific survival (DSS) was defined as the interval between the date of surgery and that of death due to endometrial carcinoma. Survival curves were plotted via the Kaplan-Meier method and compared using the log-rank test. To identify prognostic predictors, univariate and multivariate survival analyses were performed using the Cox proportional hazards regression model, and hazard ratios for recurrence and death, along with their 95% confidence intervals (CIs), were calculated. The predictive capacity (discrimination) of the molecular classification was evaluated using Harrell’s concordance index (C-index). All statistical analyses were performed using the IBM Statistical Statistics for Windows (version 20.0; IBM Corp., Armonk, NY, USA); a 2-sided *p*-value of <0.05 was considered statistically significant.

## 3. Results

This section may be divided by subheadings. It should provide a concise and precise description of the experimental results, their interpretation, as well as the experimental conclusions that can be drawn.

### 3.1. Molecular Classification in High-Grade Endometrial Carcinomas

A total of 415 patients diagnosed with high-grade endometrial carcinoma were initially identified. Patients who received neoadjuvant chemotherapy before surgery (N = 16), those with concurrent cervical cancer (N = 4) or ovarian cancer (N = 7), those with recurrent endometrial carcinomas who underwent hysterectomy and/or radiotherapy (N = 21), and those with inadequate formalin-fixed and paraffin-embedded tissue blocks (N = 12) were excluded. Thus, 355 patients with a median age of 59 years (range, 30–85 years) were ultimately included in this study, among whom 177 had EEC, 48 had serous carcinoma, 39 had clear cell carcinoma, 19 had carcinosarcoma, six had UEC/DEC, and 66 had mixed carcinomas. Of the 66 mixed carcinomas, 14 were comprised with high-grade EEC and clear cell carcinoma, 13 with high-grade EEC and serous carcinoma, 18 with low-grade EEC and clear cell carcinoma, 18 with low-grade EEC and serous carcinoma, two with serous and clear cell carcinomas, and one composed of three different histological types (high-grade EEC, clear cell carcinoma, and carcinosarcoma). The clinicopathological characteristics of the patients are summarized in Table 1.

Upon the molecular classification of the 355 patients, 42 (11.8%) were categorized into the *POLE*mut, 106 (29.9%) into the MMRd, 128 (36.1%) into the p53mut, and 79 (22.2%) into the NSMP subgroups. The distributions of molecular classifications among patients with different clinicopathological parameters are listed in Table 1. P53mut was more frequently observed among patients aged >59 years and those with advanced-stage disease (FIGO III–IV). Of 177 patients with high-grade EEC, 18.1% were categorized as *POLE*mut and 16.9% as p53mut. Most (81.3%) serous carcinomas were classified as p53mut, which was more frequently observed among patients with clear cell carcinoma (43.6%) and carcinosarcoma (63.2%). In contrast, MMRd was more common in patients with UEC/DEC. Patients with lymphovascular space invasion (LVSI) presented a higher proportion of MMRd than those without (38.6% vs. 24.2%) and a lower proportion of *POLE*mut (6.9% vs. 16.5%).

### 3.2. Multiple Molecular Alterations

As Leon-Castillo et al. described [26], we made analysis for multiple molecular alterations. A total of 24 patients (6.8%) had tumors with more than one molecular aberration. Among these, two tumors presented with three aberrations (synchronous p53mut, *POLE*mut, and MMRd) while 22 presented with two (12 with p53mut and MMRd, seven with p53mut and *POLE*mut, and three with MMRd and *POLE*mut). Among these 24 tumors, 11 were EECs, three were serous carcinoma, three were carcinosarcomas, two were clear cell carcinomas, one was a UEC/DEC, and four were mixed carcinomas.

### 3.3. Prognostic Significance of Molecular Classification among Patients with High-Grade Endometrial Carcinomas

After excluding patients for whom relevant specimens were lacking as well as those whose follow-up times were under three months, 292 patients who underwent final hysterectomy with complete adjuvant systemic therapy (chemotherapy and/or radiotherapy) were subjected to survival analysis. There were no significant differences between the entire cohort of 355 patients and the survival analysis-only subgroup in terms of clinicopathological parameters. During a median follow-up interval of 43 months (range, 8–121 months), 58 patients (19.9%) relapsed and 43 (14.7%) died of endometrial carcinoma.

**Figure 1:**
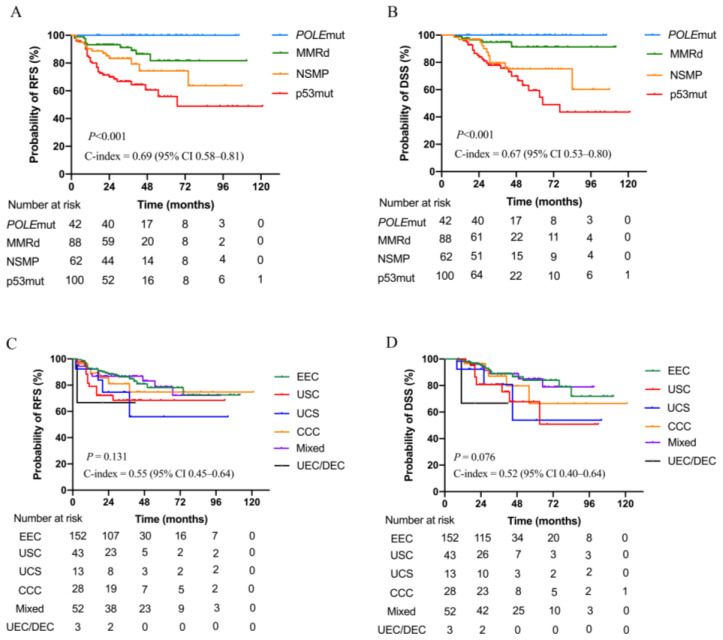
Kaplan-Meier curves showing the relapse-free survival (RFS) and disease-specific survival (DSS) of patients with high-grade endometrial carcinomas classified according to molecular subtype or histotype. (**A**) RFS stratified by molecular subtype; (**B**) DSS stratified by molecular subtype; (**C**), RFS stratified by histotype; and (**D**) DSS stratified by histotype. EEC, endometrioid endometrial carcinoma; MMRd, mismatch repair deficient; NSMP, non-specific molecular profile; p53mut, P53 mutation; *POLE*mut, polymerase epsilon mutation; UEC/DEC, undifferentiated or dedifferentiated endometrial carcinoma; USC, uterine serous carcinoma; CCC, clear cell carcinoma; USC, uterine carcinosarcoma.

**Figure 2:**
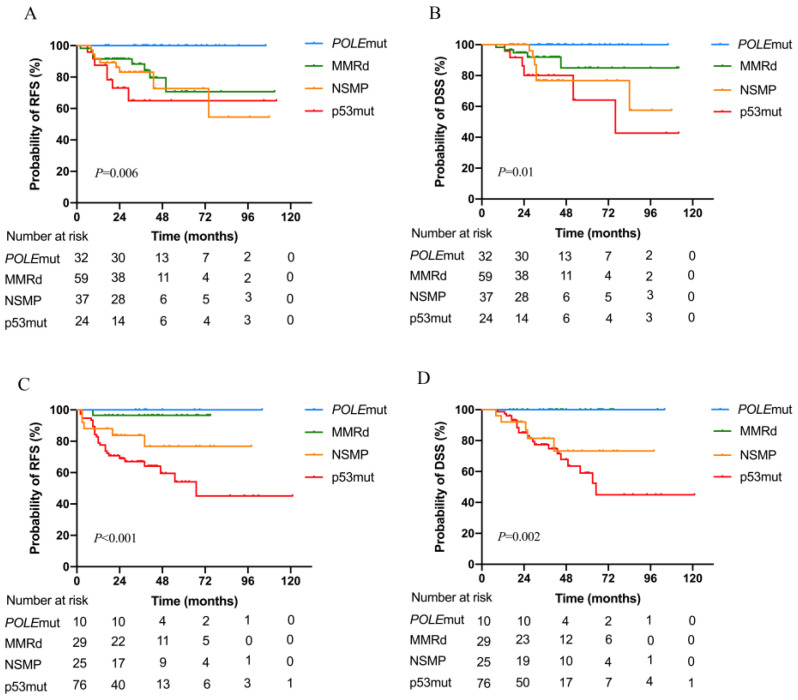
Kaplan-Meier curves showing the relapse-free survival (RFS) and disease-specific survival (DSS) of patients with high-grade endometrioid endometrial carcinoma (EEC) or non-EEC classified according to molecular subtype. (**A**) RFS in patients with EEC; (**B**) DSS in patients with EEC; (**C**) RFS in patients with non-EEC; and (**D**) DSS in patients with non-EEC. NSMP, non-specific molecular profile; p53mut, P53 mutation; *POLE*mut, polymerase epsilon mutation; UEC/DEC, undifferentiated/dedifferentiated endometrial carcinoma.

Kaplan-Meier analyses showed distinct survival curves for patients with different molecular subtypes (Figure 1A,B); the C-indices for RFS and DSS were 0.69 (95% CI 0.58–0.81) and 0.67 (95% CI 0.53–0.80), respectively. In contrast, the histopathologic subtype was not significantly associated with RFS and DSS (Figure 1C,D). Univariate analysis showed that age (≥59 vs. <59 years), FIGO stage, histology (non-EEC vs. EEC), depth of myometrial invasion, LVSI status, and molecular subgroup were significantly associated with survival outcomes (Table 2). Multivariate analysis showed that the molecular subgroup remained significantly associated with RFS and DSS independent of FIGO stage or LVSI status (Table 3). When confined to the EEC or non-EEC subtypes, molecular classification was significantly associated with RFS and DSS (Figure 2).

### 3.4. Shift of Prognostic Risk Group

Using the 2021 European Society of Gynecological Oncology (ESGO), the European Society for Radiotherapy and Oncology (ESTRO), and the European Society of Pathology (ESP) clinical practice guidelines [2], all 335 patients were stratified without incorporating molecular classification into the model and again with these classifications included. When risk groups were determined without taking molecular classifications into account, no patients fell into the low-risk group, whereas 66 (19.7%) were at intermediate risk, 67 (20.0%) at high-intermediate risk, and 171 (51.0%) at high risk. Upon incorporating the molecular classifications, 52 patients (15.5%) switched to another risk group: 40 (11.9%) shifted to a lower risk owing to being classified as *POLE*mut, while 12 (3.6%) shifted to a higher risk because of their p53mut status. A detailed comparison of the two prognostic risk groups is presented in Table 4.

We evaluated the practical application of molecular sub-typing in the management of high-grade endometrial carcinomas. For the sake of clarity and simplicity, we simply selected *POLE* and p53mut as molecular markers in grade 3 EEC, clear cell, and serous histology (N = 256). A total of 34 (13.3%) patients were classified as *POLE*mut and shifted to a lower risk, and 12 (4.7%) shifted to a higher risk owing to their p53mut status.

## 4. Discussion

Bokhman’s classification of endometrial carcinomas into estrogen-related EECs (type I) and estrogen-independent non-EEC (type II) provided a framework for attaining a better understanding of this disease. However, the characteristics of type I and type II tumors significantly overlap, and some malignancies fall into neither category, making it difficult to apply this dichotomized model in clinical practice. Risk classification of endometrial carcinoma for purposes of assessing prognosis and planning adjuvant therapy has traditionally been based on tumor grade, histology, and clinical stage; however, histotype assignment is sub-optimally reproducible in high-grade endometrial carcinoma, even among specialist gynecologic pathologists. Additionally, the histotype may not be independently associated with survival outcomes, as some non-EECs are intrinsically high-grade tumors that develop in older patients and are usually diagnosed at advanced stages. Molecular classification of endometrial carcinomas is useful and provides a more objective framework for assessing prognosis and planning adjuvant treatment. Such classifications have been applied to endometrioid and serous carcinomas; however, little is known about the relevance of this framework to high-grade endometrial carcinomas. To our knowledge, ours is the first study to investigate the molecular classification in a large cohort of patients with high-grade endometrial carcinomas, including EEC and non-EEC histotypes. Our results indicated that the molecular subtype was independently associated with clinical outcomes whereas the histotype was not. Our data thus indicate that molecular classification could provide prognostic and predictive information beyond traditional clinicopathological variables and suggest that the combined assessment of molecular characteristics and clinicopathological features in patients with high-grade endometrial carcinomas may improve risk stratification and therapeutic decision-making.

The molecular subtype distribution in grade 3 EEC has been described in two previous studies [12,13]. One included 381 patients with grade 3 EEC from six institutions and found that 12.9% had *POLE*mut, 36.2% had MMRd, 30.2% had NSMP, and 20.7% had p53mut [13]. The other study included 95 patients with grade 3 EEC from a single institution and found that 11% presented with *POLE*mut, 37% with MMRd, 27% with NSMP, 19% with p53mut, and 6% with multiple classifier [12]. Our present study included 177 patients with high-grade EEC exhibiting distributions that were consistent with the two aforementioned studies: *POLE*mut, 18.1%; MMRd, 39.5%; NSMP, 25.4%; and p53mut, 17.0%. Our patients also experienced distinct survival outcomes depending on their tumors’ molecular subtype, which was consistent with data from Bosse et al.’s multi-institutional study [13]. These results suggest that molecular classification can segregate clinical outcomes and assess the prognoses of patients with grade 3 EEC.

Clear cell carcinoma is a rare histotype of endometrial cancer, and little is known about its molecular distribution. Kim et al. analyzed 52 patients with clear cell carcinoma and identified five (9.6%) with MMRd, one (1.9%) with *POLE*mut, 28 (53.8%) with NSMP, and 18 (34.6%) with p53mut [14]. A meta-analysis of 162 patients with clear cell carcinoma from five studies found that the most prevalent subgroups were p53mut (42.5%) and NSMP (40.9%), while the MSI/MMRd (9.8%) and *POLE*mut (3.8%) were less common [27]. Our present study included 39 patients with clear cell carcinoma among whom the most prevalent subtype was p53mut (43.6%), whereas 23.1% had MMRd and 28.2% had NSMP. These data suggest that clear cell carcinoma is a heterogeneous disease with the characteristics of all four molecular subtypes, rather than a homogeneous group.

Uterine carcinosarcoma is a rare but highly aggressive tumor that comprises malignant epithelial and stromal components. The epithelial component is most often a serous carcinoma and grade 3 EEC. From a molecular point of view, approximately 80% of these tumors share common molecular aberrations with serous carcinoma [28]. The present study included 19 patients with carcinosarcomas; 63.2% showed p53mut, 10.5% had MMRd, and 10.5% harbored *POLE*mut. In a pooled analysis of 231 patients from four studies, Travaglino et al. found that the distribution of the molecular classifications was 5.3% with *POLE*mut, 7.3% with MSI/MMRd, 73.9% with p53mut, and 13.5% with NSMP [15]. Although the majority of carcinosarcomas present with p53mut, a subset of them harbored *POLE*mut or MMRd, indicating that these patients have favorable survival outcomes and may benefit from immune checkpoint inhibitor therapies.

Previous studies involving small cohorts have revealed that molecular classification is also applicable to non-EEC subtypes [14,15,16]. The present study included 178 patients without EEC, accounting for 51.1% of the cohort, and our data showed that patients in different molecular subgroups experienced distinct clinical outcomes. Hence, our data (which were from a single-institution study with a large sample size) also demonstrated that molecular classifications can be applicable to non-EEC subtypes. Compared with the 2016 guidelines for the management of patients with endometrial carcinoma [29], the highlight of the 2021 ESGO/ESTRO/ESP guidelines was risk group assignment based on molecular classification [2]. Adjuvant treatment is usually recommended for high-grade and/or high-risk endometrial carcinomas; thus, molecular classification appears to be particularly useful for these patients [2]. In this study, we compared risk groupings using models in which the molecular classifications were either incorporated or not; the risk group of 15.5% of the patients changed when these classifications were applied, with 11.9% and 3.6% shifting to lower- and higher-risk categories, respectively. In an unselected cohort of 594 patients, including 166 with high-grade endometrial cancers, Imboden et al. demonstrated that 3.7% of high-risk patients shifted toward a lower risk owing to *POLE*mut while 2.9% of low-risk patients shifted toward a higher risk owing to p53mut [30]. Compared with results from the unselected cohort, a greater proportion of patients with high-grade endometrial carcinomas showed a downward shift due to *POLE*mut (11.9% vs. 3.7%). Additionally, 42 patients in our cohort with *POLE*mut had excellent prognoses, and none experienced disease relapse. Without molecular classification, these patients would receive adjuvant treatment; however, omission of adjuvant treatment should be considered for patients with stage I–II low-risk disease based on the presence of pathogenic *POLE*mut [2]. The sizable number of patients with high-grade endometrial carcinomas whose risk assessment shifted when molecular classification was incorporated indicates that clinical management could be significantly impacted; thus, molecular classification of these patients is highly warranted. No adjuvant treatment should be considered for patients with high-grade endometrial carcinomas exhibiting *POLE*mut molecular subtype. Combined post-operative radiotherapy and systemic chemotherapy could be considered for patients with p53mut subtype.

Our study had some limitations. First, this was a retrospective investigation that produced inherent and unavoidable biases. Second, the small number of patients representing each specific histotype limited the subgroup survival analysis. Lastly, our study was restricted to a single institution and lacked an independent validation cohort. Additional studies using independent cohorts are needed to demonstrate the impact of molecular classification on the outcomes of patients with high-grade endometrial carcinomas.

## 5. Conclusions

Our findings showed that molecular subtype, but not histotype, was independently associated with survival outcomes in patients with high-grade endometrial carcinomas, and that molecular classification was applicable to patients with both high-grade EEC and non-EEC subtypes. Our data suggested that incorporating molecular classification status into clinicopathological assessment in patients with high-grade endometrial carcinomas may provide more accurate prognostic information and better stratification vis-à-vis clinical management.

## Figures and Tables

**Table 1:** Molecular classification of patients with high-grade endometrial carcinoma (N = 355) distributed according to their clinicopathological parameters.

		Molecular Classification				
Parameters	N (%)	*POLE*mut	MMRd	NSMP	p53mut	*p*-Value
	355	42 (11.8)	106 (29.9)	79 (22.3)	128 (36.1)	
Age						<0.001
≤59 years	179 (50.4)	28 (15.6)	68 (38.0)	45 (25.1)	38 (21.2)	
>59 years	176 (49.6)	14 (8.0)	38 (21.6)	34 (19.3)	90 (51.1)	
FIGO stage (N = 335)						<0.001
I	184 (54.9)	36 (19.6)	56 (30.4)	44 (23.9)	48 (26.1)	
II	22 (6.6)	3 (13.6)	8 (36.4)	7 (31.8)	4 (18.2)	
III	99 (29.6)	1 (1.0)	31 (31.3)	17 (17.2)	50 (50.5)	
IV	30 (9.0)	0 (0.0)	8 (26.7)	5 (16.7)	17 (56.7)	
Histology						
Grade 3 EEC	177 (49.9)	32 (18.1)	70 (39.5)	45 (25.4)	30 (17.0)	<0.001
Serous carcinoma	48 (13.5)	1 (2.1)	4 (8.3)	4 (8.3)	39 (81.3)	
Clear cell carcinoma	39 (11.0)	2 (5.1)	9 (23.1)	11 (28.2)	17 (43.6)	
Carcinosarcoma	19 (5.4)	2 (10.5)	2 (10.5)	3 (15.8)	12 (63.2)	
UEC/DEC	6 (1.7)	0 (0.0)	4 (66.7)	1 (16.7)	1 (16.7)	
Mixed carcinoma	66 (18.6)	5 (7.6)	17 (25.8)	15 (22.7)	29 (43.9)	
Myometrial invasion (N = 338)						0.049
<1/2	184 (54.4)	31 (16.8)	54 (29.3)	40 (21.7)	59 (32.1)	
≥1/2	154 (45.6)	11 (7.1)	49 (31.8)	33 (21.4)	61 (39.6)	
LVSI (N = 339)						0.006
Absent	194 (57.2)	32 (16.5)	47 (24.2)	45 (23.2)	70 (36.1)	
Present	145 (42.8)	10 (6.9)	56 (38.6)	28 (19.3)	51 (35.2)	

EEC, endometrioid endometrial carcinoma; FIGO, International Federation of Gynecology and Obstetrics; LVSI, lymphovascular space invasion; MMRd, mismatch repair deficient; NSMP, non-specific molecular profile; p53mut, P53 mutation; *POLE*mut, polymerase epsilon mutation; UEC/DEC, undifferentiated/dedifferentiated endometrial carcinoma.

**Table 2:** Univariate analysis of factors potentially predictive of survival in patients with high-grade endometrial cancer.

Parameters	Relapse-Free Survival		Disease-Specific Survival	
	HR (95% CI)	*p*-Value	HR (95% CI)	*p*-Value
Age (years)				
≥59 vs. <59	1.61 (0.95–2.72)	0.074	2.01 (1.08–3.74)	0.029
FIGO stage				
II–IV vs. I	4.41 (2.41–8.08)	<0.001	4.59 (2.25–9.34)	<0.001
Histology				
Non-EEC vs. EEC	1.57 (0.93–2.65)	0.090	1.63 (0.88–3.01)	0.117
Myometrial invasion				
≥1/2 vs. <1/2	1.51 (0.90–2.55)	0.119	1.99 (1.08–3.70)	0.028
LVSI				
Present vs. absent	1.78 (1.05–3.01)	0.032	2.66 (1.42–4.96)	0.002
Molecular subtype		0.003		0.007
*POLE*mut vs. NSMP	0.0 (0.0–2.3 × 10^200^)	0.956	0.0 (0.0–1.4 × 10^232^)	0.961
MMRd vs. NSMP	0.54 (0.23–1.20)	0.125	0.33 (0.11–0.95)	0.039
p53mut vs. NSMP	1.89 (1.00–3.58)	0.050	1.71 (0.85–3.45)	0.134

CI, confidence interval; EEC, endometrioid endometrial carcinoma; FIGO, International Federation of Gynecology and Obstetrics; HR, hazard ratio; LVSI, lymphovascular space invasion; MMRd, mismatch repair-deficient; NSMP, non-specific molecular profile; p53mut, P53 mutation; *POLE*mut, polymerase epsilon mutation.

**Table 3:** Multivariate analysis of factors potentially predictive of survival in patients with high-grade endometrial cancer.

Parameters	Relapse-Free Survival		Disease-Specific Survival	
	HR (95% CI)	*p*-Value	HR (95% CI)	*p*-Value
FIGO stage				
II–IV vs. I	3.03 (1.64–5.64)	<0.001	2.38 (1.09–5.16)	0.029
Molecular subtype		<0.001		<0.001
*POLE*mut vs. NSMP	0.0 (0.0–1.7 × 10^221^)	0.961	0.0 (0.0–1.9 × 10^200^)	0.966
MMRd vs. NSMP	0.52 (0.23–1.21)	0.129	0.28 (0.09–0.82)	0.021
p53mut vs. NSMP	1.59 (0.82–3.10)	0.169	1.40 (0.67–2.94)	0.370
LVSI				
Present vs. absent			2.07 (1.05–4.06)	0.035

CI, confidence interval; FIGO, International Federation of Gynecology and Obstetrics; HR, hazard ratio; LVSI, lymphovascular space invasion; MMRd, mismatch repair deficient; NSMP, non-specific molecular profile; p53mut, P53 mutation; *POLE*mut, polymerase epsilon mutation.

**Table 4:** Concordance and shift between risk groups when considering vs. not considering molecular classification status.

	Molecular Classification Considered					Total (N, %)
Molecular Classification Not Considered	Low	Intermediate	High–Intermediate	High	Advanced Metastatic	
Intermediate	18 ↓	43	0	5 ↑	0	66 (19.7)
High–intermediate	16 ↓	0	44	7 ↑	0	67 (20.0)
High	6 ↓	0	0	165	0	171 (51.0)
Advanced metastatic	0	0	0	0	31	31 (9.3)
Total (N, %)	40 (11.9)	43 (12.8)	44 (13.1)	177 (52.8)	31 (9.3)	335

↓ indicates a downward shift in risk while ↑ indicates an upward shift.

## Data Availability

The raw data used in this study are available from the corresponding author upon reasonable request.
